# Improvement of EEG Signal Acquisition: An Electrical Aspect for State of the Art of Front End

**DOI:** 10.1155/2010/630649

**Published:** 2010-02-02

**Authors:** Ali Bulent Usakli

**Affiliations:** Department of Technical Sciences, The NCO Academy, 10100 Balikesir, Turkey

## Abstract

The aim of this study is to present some practical state-of-the-art considerations in acquiring satisfactory signals for electroencephalographic signal acquisition. These considerations are important for users and system designers. Especially choosing correct electrode and design strategy of the initial electronic circuitry front end plays an important role in improving the system's measurement performance. Considering the pitfalls in the design of biopotential measurement system and recording session conditions creates better accuracy. In electroencephalogram (EEG) recording electrodes, system electronics including filtering, amplifying, signal conversion, data storing, and environmental conditions affect the recording performance. In this paper, EEG electrode principles and main points of electronic noise reduction methods in EEG signal acquisition front end are discussed, and some suggestions for improving signal acquisition are presented.

## 1. Introduction

Although basics of the electroencephalogram (EEG) measurement in man have been the same since 1929, it was first made by Hans Berger, the technological developments give the opportunity to build much more sophisticated acquisition systems regarding clinical needs and scientific researches. The human brain generates electrical signals called EEG signals which are related to body functions, and this paper is about their acquiring. These signals are roughly less than 100 *μ*V and 100 Hz and can be measured with electrodes placed on the scalp, noninvasively. Because of their low amplitude due to the skull's composition, the measurement of EEG is more difficult than the other noninvasive biosignal measurements such as the electrocardiogram, electromyogram, electrooculogram, and so forth. Having expensive bio-signal recording systems cannot guarantee acquiring proper signals. In that sense, some factors to acquire good EEG signals should be considered in new designs and during recording sessions. These major considerations are discussed and some suggestions are presented in this paper.

**Figure 1:**
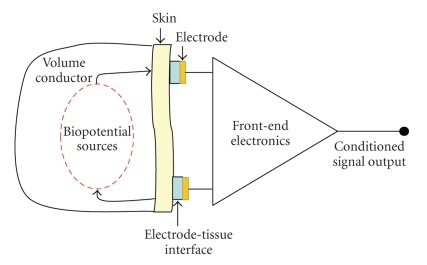
Biopotential measurement via electrodes.

In bio-signal recordings, electrodes are the initial elements which are used for converting biopotential signals due to biopotential sources into electrical signals. Figure 1 shows the simplified biopotential measurement. EEG electrodes are usually made of metal and are produced as cup-shaped, disc, needle, or microelectrode to measure intra-cortex potentials. Silver chloride (AgCl) is preferred for common neurophysiologic applications [1]. Because Ag is a slightly soluble salt, AgCl quickly saturates and comes to equilibrium. Therefore, Ag is a good metal for metallic skin-surface electrodes [2]. Choosing the correct electrode as well as preparation of the skin before recording affects the accuracy of the measurements.

Another major factor is electronic noise which is quite important for the bio-signal measurements. Electronic noise can be caused by internal and external noise sources. The internal noise sources are thermal (due to resistive components), shot (due to semiconductor holes and diffusions), flicker (due to contact pins), and burst (or popcorn, due to impurities in semiconductors) noise [3]. The most important external noise is caused by power-line interference. It is clearly seen in spectral analysis at 50 Hz (or 60 Hz). Between power lines and the subject there are capacitances (parasitic and isolation). To extract biosignals precisely from electronic noise requires efficient noise reduction methods [3, 4]. Efficient analog and/or digital filtering are needed for this purpose. 

In the following sections, EEG electrodes as well as EEG recording and design considerations are presented.

## 2. Materials and Methods

### 2.1. EEG Electrodes

Electrodes may be polarized (nonreversible) or nonpolarized (reversible). Polarization is avoided since the chloride ion is common to both the electrode and the electrolyte. Other metals such as gold or platinum can be used for electrode fabrication but is costly. Polarized electrodes tend to make significant capacitance, and this may interfere with the transmission of underlying bio-signals. These electrodes behave like a low-frequency filter (low-pass filter). Non-polarized electrodes, such as those of AgCl, are preferred for common neurophysiologic applications [1, 2]. Normal Ag/AgCl electrodes need to be chlorinated in time; however, sintered (making electrodes from powder, by heating the material in a sintering furnace below its melting point) Ag/AgCl electrodes do not need to be chlorinated.

The EEG electrodes can be classified as disposable reusable disc and cup shaped (EEG caps), subdermal needles (single-use needles that are placed under the skin), and implanted electrodes (to precisely pinpoint the origin of seizure activity). Needles are available with permanently attached wire leads, where the whole assembly is discarded, or sockets that are attached to lead wires with matching plugs. They are made of stainless steel or platinum. Some of EEG electrodes can be used for special applications. For example, implanted EEG electrodes also can be used to stimulate the brain and map cortical and subcortical neurological functions, such as motor or language function, in preparation for epilepsy surgery. Infection must be considered a major risk of implanted EEG electrodes.

In a noninvasive electrical brain signal measurement, there is an interface material between the electrode and the skin. This material is an electrolyte and can be in EEG gel or paste form. The electrophysiological activity caused by a biopotential source is a current source that causes current flow in the extracellular fluid and other conductive paths through the tissue.

**Figure 2:**
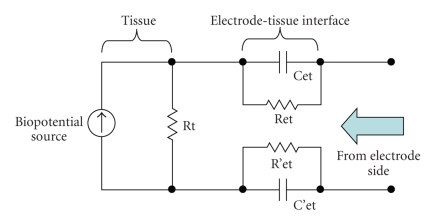
Simplified equivalent circuit of biopotential source and electrode-tissue interface from electrode. Biopotential source as a current source and tissue resistance is shown Rt. Cet and Ret electrode-tissue equivalent elements may change for each electrode contact.

A cup-shaped electrode provides enough volume to contain an electrolyte, including chlorine ions. In these electrodes, the skin never touches the electrode material directly. The electrode-tissue interface has impedance depending on several factors. Some of these factors are the interface layer (such as skin preparation, fat mass, hair, etc.), area of electrode's surface, and temperature of the electrolyte. The electrode-tissue contact can be modeled as in Figure 2. As it is seen from the figure, the electrode-tissue interface not only is resistive but consists of capacitive elements too. This is important for the frequency dependency of the electrode-skin contact.

Because of the interaction between metallic electrode and electrolyte, the ions accumulated as parallel plates. Ion-electron exchange occurs between the electrode and the electrolyte. This exchange results in voltage and it is called the half-cell potential. Because of this potential, in some cases, biopotential amplifiers must tolerate up to ±300 mV. This value depends on the electrode and electrolyte materials. This can be explained by the Nernst Equation, simply, as

(1) $$\varepsilon =\varepsilon^{0}-\frac{0,05916}{n_{e}}\log\;\;Q.$$

Here, we have *ε*: Half-cell potential (V), *n*_*e*_: Transported electron (mol number), and *Q*: Rate of inside and outside ions: *Q* = [Ions_Inside_]/[Ions_Outside_].

In clinical EEG recordings, 10/20 Electrode Placement System is a standard and in general, it has been used for many clinical or research applications [5]. Although there are 75 locations in this system, 8 to 32 electrodes may be sufficient for clinical applications. 8 channels can also be sufficient for some Brain Computer Interface (BCI) applications; on the other hand, for Electrical Source Imaging (ESI) more than 100 channels are required. Electrodes are positioned over the frontal, temporal, parietal, and occipital lobes, and odd and even numbers refer to the left and right hemispheres, respectively. Because of the requirements, another placement system is 5% electrode placement and 345 locations are determined [6], but it is not a common standard.

### 2.2. EEG Recordings

In the EEG system, as a non-invasive application, the electrodes are placed on the scalp, and a sufficient number of electrodes may be 1 to 256 (or more in near future) placed on EEG cap for easy application. To provide ionic current and to reduce contact impedance between the electrode surface and the scalp, EEG gel or paste must be used together for proper skin preparation. In biopotential measurements, the most important point is preserving the biosignal's originality. The contact impedance should be between 1 kΩ to 10 kΩ to record an accurate signal. Less than 1 kΩ contact impedance indicates a possible shortcut between electrodes; on the other hand, impedance greater than 10 kΩ can cause distorting artifacts.

Drying the gel or paste in time, as well as the subject's perspiration and movements (eye blinks, muscle movements, heart beats, etc.), can easily affect the measurement performance negatively. Because of these reasons, recording time is generally limited for several hours. For long periods of time or ambulatory EEG recordings, additional requirements are necessary to make patients more comfortable and to allow for consistent system performance. High-resolution applications such as ESI or wireless data transfer also require a different approach for the design of the novel electrodes. To reduce the skin preparation time and to measure the bio-signals more accurately, several approaches are attempted for electrode fabrication. For example, multiarray thin film electrodes are developed especially for different depth in operational applications [10]; nitride-covered steel is used as an electrode and there is no need for EEG paste to result in successful recordings [11]. In the last few years, active electrode (small or unity gain amplifier close to electrode) research is gaining popularity. With these types of electrodes, without the use of electrode gel and with much more skin preparation, noise reductions are reported [9, 12–14].

**Figure 3:**
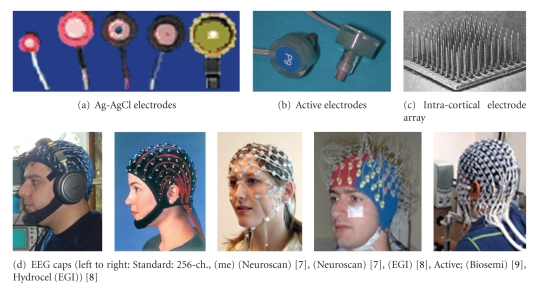
Commercially available EEG electrodes and cap samples; (c) is for invasive applications.

In commercial applications, apart from classical cup- or disc-shaped electrodes and active electrodes, another approach is used to reduce preparation time (by EGI's HydroCel Geodesic Sensor Net). In this approach, scalp preparation and abrasion are not required. Because the soft pedestal design of the chamber creates a sealed environment, it hydrates the skin and creates an interface between the skin and electrode. Application times for the sponge-based HydroCel Geodesic Sensor Net that range between 5 minutes for 32 channels to 15 minutes for 256 channels are reported [8]. In practical consideration, at least 45 minutes are required for the electrode while 15 minutes are reasonable in skin preparations for the 256-channel cup-shaped electrode cap. Figure 3 shows some EEG electrodes and caps commercially available. In this figure several examples as non-invasive electrodes and EEG caps as well as one intracortical electrode array are shown.

**Figure 4:**
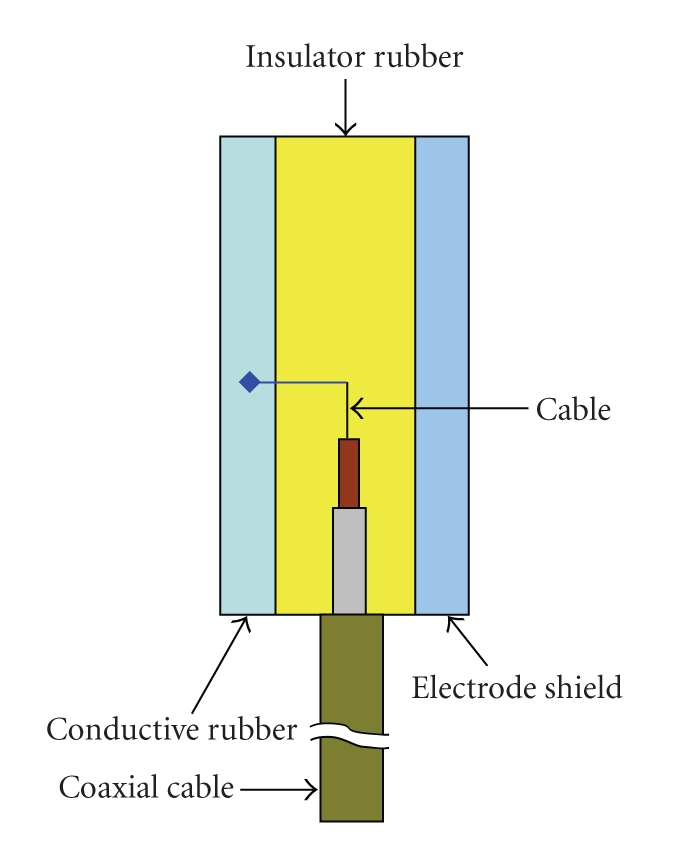
A dry electrode principle.

Another approach for fabricating EEG electrode is dry electrode (Figure 4). This type of electrode does not need an extensive set-up time, and it is convenient for long-term recordings. These properties are advantageous for BCI and neurofeedback applications. As an example, in order to design dry electrode, 1.5 mm thick silicone conductive rubber-shaped discs of 8 mm diameter are used. The active side of the electrode is capacitive and coupled through a layer of insulating silicon rubber with a metal shield wired to the active guard shield. The impedance of the realized electrodes at 100 Hz is greater than 20 MΩ with a parasitic capacitance smaller than 2 pF [15].

For under cortex applications intra-cortical electrodes are used. One of these types of electrodes (The Utah Intra-cortical Electrode Array) is an array of 100 penetrating silicon microelectrodes designed to electrically focus stimulation or record neurons residing in a single layer up to 1.5 mm beneath the surface of the cerebral cortex [16]. Each electrode of the intra-cortical array electrode is 1.5 mm long, 0.08 mm wide at the base, and 0.001 mm at the tip.

**Figure 5:**
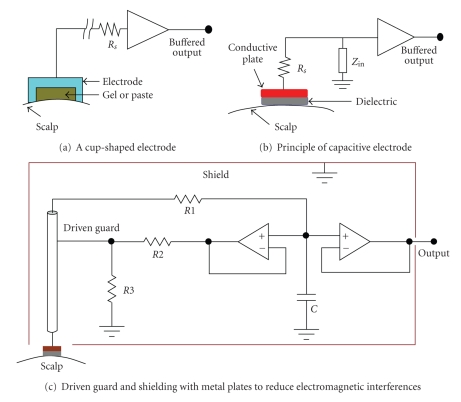
EEG electrode connections.

Each type of electrode should be used with a successful electronic circuit. In Figure 5 EEG electrode and initial signal acquisition examples are shown. Recording environment conditions, contact impedance value and its stability, amplification method (ac or dc), and recording time must be considered. In the next subsection design considerations are explained, briefly.

### 2.3. The Design Considerations

**Figure 6:**
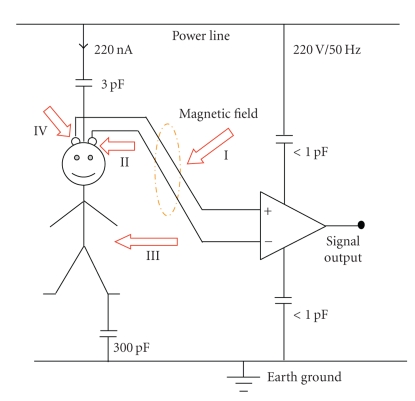
Electromagnetic interference (EMI) ways (for the capacitance values [9]). Arrows show the interference currents. (I) Voltage due to magnetic field to electrode cable loop is illustrated. (II) Displacement current on subject head due to electrical field causes voltage drop across electrodes. (III) Displacement current on subject body due to electrical field causes voltage drop across electrodes. (IV) Additionally, this current causes voltage between measurement electrode and amplifier common pin [24].

In EEG recordings, as the other bio-signal measurements, one of the major problems is the 50 (or 60) Hz noise due to power lines. Between power lines and the subject there are capacitances (parasitic and isolation). Electromagnetic interference (EMI) ways are shown in Figure 6. The environmental factors influence the subject and measurement system. For example, a fluorescent lamp 1-2 m away from the measurement system interferes with the measuring signal as several kHz peaks. The interference signal may be half of the power line noise. In the same way, other electrical or electronic devices may interfere with the bio-signal measurements.

The dc component of the common mode signal is about several thousand, volts and the ac component may be about 1 V. This value may be in mV scale when the subject's body is grounded with earth ground and may be as high as 20 V when power line is held [17]. Electrostatic discharge (ESD) and defibrillator should be considered in electronic design. Protection for these must be provided for patient/subject and also initial active components. To reduce common mode signal effects, the instrumentation amplifiers having higher common mode rejection ratio (CMRR) must be used [18, 19]. Some research studies related to biopotential design report that 80–136 dB CMRRs are obtained [20–23]. To reduce isolation capacitance effects, battery powered operation is efficient [9].

In order to guarantee the subject/patient safety, leakage current should be less than the levels determined by IEC 601-1. According to this regulation, leakage current must be less than 10 *μ*A in normal conditions, while regarding the connection to the main power supply, 50 *μ*A is allowable.

Biopotential amplifiers can be dc coupled or ac coupled. In design strategy, dc or ac coupling and filtering (hardware or software) decisions are the initial steps for the biopotential measurement system designer. For ac amplification more than 10 bits digital resolution may be enough. However, for dc amplification, because number of effective bits is decreased, more than 20 bits are necessary for the analog digital converter (ADC). For high digital resolution sigma-delta technology ADC is one of good solutions [22, 23].

**Figure 7:**
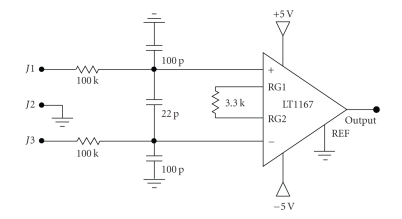
Suggested input amplifier circuit.

The input amplifier circuit is presented in Figure 7. It can be used wired electrodes (classical approach) or close to electrodes (active electrode approach). After the tests, it is observed that the circuit can be used for EEG, EOG or EMG signal measurements. This amplifier's gain is 16, and it does not cause biopotential amplifier saturation, and its CMRR is 102 dB under no shielding conditions or EMI protection.

## 3. Discussions

Acquiring EEG signal properly means mainly safety, bio-signal measurement with higher Signal to Noise Ratio (SNR) and no data loss. The major points that the author briefly proposed for the entire recording process are the following.

(i)* Subject/Patient Safety*. Because of the leakage current from system electronics and defibrillator (if used), subject/patient safety should be provided. Subject/patient and front-end circuitry and earth grounds should be separated (i.e., analog and digital grounds). Increasing the isolation mode rejection ratio of the amplifier reduces the influence of isolation mode voltage.

(ii) *EMI Protection*. Operation of electrical or electronic devices and especially fluorescent lamps near the recording set-up is prohibited. Otherwise acquired EEG signals are distorted and the signal corrupted with noise. Using instrumentation amplifier can help getting rid of this problem.

(iii) *No Subject/Patient Muscular Movements*. Muscular movements (i.e., EMG-related contamination) such as eye blinks, clenching teeth, movement of shoulders or legs, and so forth affect EEG signal acquisition, badly. These cases may cause wrong comments on the signals and signal processing error.

(iv) *ESD Protection*. Active electronic components must have greater than 2000 V ESD protection. No ESD protection may cause damage of active electronic components and may cause serious problem for subject/patient. 

(v) *Efficient Grounding*. Metal cases must be connected to metal plate/rod buried under ground. Proper grounding technique helps to reduce noise therefore increasing SNR.

(vi) *Electrodes*. Choosing correct electrode and montage should be decided regarding clinical or research application purposes. In addition to availability of commercial standard or active types, electrodes can be made such as capacitive coupling or dry electrode. Number of electrodes and their placement is also important for the application.

(vii) *Electrode Contact Impedance*. Contact impedance value must be between 1 kΩ and 10 kΩ for classical electrodes. Less than 1 kΩ contact impedance indicates probable shortcut between the electrodes. Greater than 10 kΩ contact impedance prevents acquiring EEG signals. Before measurement, contact impedance should be measured, and EEG trace should be observed while recording. Using todays technology, high input impedance (>1 GΩ) amplifier chips and active electrode approaches decrease dependency of the contact impedance. To acquire proper signal, electrodes should not be moved. Otherwise it causes fluctuation of the EEG signal, and spikes on it.

(viii) *Noise Immunity*. Noise reduction techniques must be considered in electronic circuitry and printed circuit board design. Electronic cards and connection cables should be placed in a metal box to reduce electronic noise as much as possible. Using twisted, blended, and driven signal cables gives good results. Because EEG signals are low amplitude *μ*Vs, they are very sensitive to electronic noise. Electronic noise should be less than 2 *μ*V (peak-to-peak). 

(ix) *Environmental Conditions*. If the EEG system is combined with magnetic resonance imaging, it must be compatible for operating under a high magnetic field. Similarly, if the EEG system is used in an operation room while surgery, it should not be affected under high electronic noise conditions such as electro-cautery. Recording must be stable and ambient temperature should not affect system performance. System performance should be independent of reasonable temperature fluctuations.

(x) *Reduction for Common Mode Signals*. To reduce common mode signals, it is necessary to use instrumentation amplifier having greater than 80 dB CMRR. This is important for high SNR signal acquisition.

(xi) *Recording Mode*. Designer or user should decide the recording mode regarding their applications. EEG recordings can be bipolar (differentiated two close electrode signal), unipolar (differentiated common reference), or using averaged reference. Clinical or research applications require different recording modes.

 (xii) *Reference and Ground Electrode Position*. Especially reference electrode placement is important for some applications. In general reference electrode is placed on vertex; ground electrode is placed on left or right (or together) ear.

(xiii) *Preserving the Biosignal Originality*. Linear and distortionless amplification must be provided. Otherwise signal processing (detection, pattern recognition, feature extraction, classification, and averaging, etc.) performances may decrease.

(xiv) *Avoiding Amplifier Saturation*. If the amplifier circuits saturate, analog signal loss is inevitable. Amplifier saturation is caused by high input signal, mainly due to electrode movements. In ac amplification, amplifier which is used before high-pass filtering must be fixed for optimum gain to avoid saturation level. If dc amplification is preferred, there is no amplifier saturation risk; on the other hand, number of efficient bit resolution is decreased.

(xv) *Cross-Talk Rejection*. For multichannel systems, cross-talk rejection must be high enough. Otherwise, channels can be affected from other channels; therefore, artifact exists.

(xvi) *Input Impedance*. Input impedance of the circuit must be high enough. For dc signals greater than 1 GΩ input impedance value gives good results. Low input impedance causes load of bio-signal source, and it causes damaging of the signal.

(xvii) *Input Bias Current*. Input bias current of the input amplifier must be as low as possible (pA). If bio-signal sources are loaded by input amplifier, it also causes distortion.

(xviii) *Frequency Band*. Selecting the proper filter band (at least band width must be 0.5 Hz–70 Hz) is important to acquire signal. This is also important for digitizing and data storing. Sufficient (and optimum) sampling rate (>140 Hz) and transfer rate should be provided. Dc level should be removed for efficient signal conditioning/processing in hardware or in software.

(xix) *Digitization*. Sufficient (and optimum) digital resolution (>10 bits for ac amplification, >20 bits for dc amplification) must be provided for analog to digital converter (ADC). If low digital resolution is used, the quantization error increases.

(xx) *Same Sampling Instants*. If the multichannel system is designed (or used), there must be no time delay between channels. For an analog multiplier, this may be a problem, however, not for a digital multiplier. To sample at the same time instants, sample and hold circuits should be used. If each analog channel has its own ADC, this can be made with ADC control signal timing.

(xxi) *Recording Time*. Sufficient (and optimum) recording time is necessary. Long-time recording (more than 2 hours) causes artifacts due to drying gel, perspiration and creating of anxiety in subject/patient. On the other hand, insufficient recording time causes insufficient data acquisition.

(xxii) *User Friendly*. The system hardware and software must be well integrated and must have user friendly interface.

(xxiii) *Low Power Consumption*. This is important for especially battery-powered systems.

(xxiv) *Low Cost*. The system should be cost effective, and components must be available.

## 4. Conclusion

There are major points that should be considered to improve the measurement performance in the design of the bio-signal measurement system or recording session. Specifically choosing the correct electrode, skin preparation, and reduction of power line noise are the important issues for EEG recordings. To reduce electromagnetic interferences, a metal box for electronic circuits, a shielded (Faraday cage principle) recording room, and guarding (driven or not) for common mode signal reduction are the efficient methods. The performance of the bio-signal measurement system depends on the electrodes, electronic circuitry, and recording conditions. Choosing the correct electrode and successful electronic design strategy are essential to acquire EEG signals, properly.
